# Transcriptome Analysis Revealed a Cold Stress-Responsive Transcription Factor, *PaDREB1A*, in *Plumbago auriculata* That Can Confer Cold Tolerance in Transgenic *Arabidopsis thaliana*

**DOI:** 10.3389/fpls.2022.760460

**Published:** 2022-03-04

**Authors:** Wenji Li, Suping Gao, Ting Lei, Liqiong Jiang, Yifan Duan, Zian Zhao, Jiani Li, Lisha Shi, Lijuan Yang

**Affiliations:** ^1^College of Landscape Architecture, Sichuan Agricultural University, Chengdu, China; ^2^Chengdu Academy of Agriculture and Forestry Sciences, Chengdu, China

**Keywords:** *Plumbago auriculata*, cold stress, transcriptome, C-repeat-binding factors, transgenic *Arabidopsis*

## Abstract

The tropical plant *Plumbago auriculata* can tolerate subzero temperatures without induction of apoptosis after cold acclimation in autumn, making it more cold tolerant than conventional tropical plants. In this study, we found that low temperatures significantly affected the photosynthetic system of *P. auriculata*. Using transcriptome sequencing, *PaDREB1A* was identified as a key transcription factor involved in the response to cold stress in *P. auriculata*. This transcription factor may be regulated by upstream JA signaling and regulates downstream *ERD4* and *ERD7* expression to resist cold stress. Overexpression of *PaDREB1A* significantly enhanced freezing resistance, protected the photosynthetic system, and enhanced the ROS scavenging mechanism under cold stress in *Arabidopsis thaliana*. Additionally, *PaDREB1A* significantly enhanced the expression of *CORs* and *CAT1* in *A. thaliana*, which further activated the downstream pathway to enhance plant cold tolerance. This study explored the possible different regulatory modes of *CBFs* in tropical plants and can serve as an important reference for the introduction of tropical plants to low-temperature regions.

## Introduction

According to the latest assessment report of the Intergovernmental Panel on Climate Change (IPCC), global climate change is intensifying. The frequent occurrence of extreme weather events, such as typhoons, heat waves, cold waves, and droughts, poses a severe threat to the vegetation in urban green spaces. With increasing globalization, urban dwellers are demanding more diverse urban green space vegetation (Hanson et al., 2021), and an increasing number of beautiful tropical plants are being introduced into urban green spaces. However, extreme weather, especially extremely low temperatures, poses a great threat to the survival of these tropical plants.

The photosystem is extremely sensitive to low temperatures and is an important indicator of whether plants have been subjected to cold stress (Chassot et al., 2001; Bilska and Sowiński, 2010). Cold stress has significant effects on the chloroplast structure, photosynthetic pigment content, photosynthetic rate, and other important physiological and biochemical parameters in plants. Photosynthesis in plants is severely impeded under cold stress. The main manifestation of this is inhibition of the biosynthesis of chlorophyll and the enzymatic activity associated with photosynthesis, leading to weakened photosynthesis. Under cold stress, the non-photochemical quenching (NPQ) of plants increases (Kramer et al., 2004; Ozturk et al., 2013), which hinders the heat dissipation pathway of photosystem II (PS II), resulting in the absorption of light energy by photosynthetic pigments far exceeding its consumption and resulting in photoinhibition (Liang et al., 2009). Whether photoinhibition occurs or not is often used as the evaluation standard of cold tolerance in plants (Long et al., 1994). The photosynthetic rate of most plants is significantly reduced by cold stress. Cold stress also affects chloroplast thylakoid membranes and reduces photochemical efficiency (Strauss et al., 2006). In addition, cold stress increases the accumulation of reactive oxygen species (ROS) in plants; ROS cause oxidative damage to DNA and proteins, damage the membrane structure, and decrease the fluidity of the cell membrane, affecting the exchange of information and materials. Cold stress also leads to the production of large amounts of malondialdehyde (MDA), which has cytotoxic effects (Neill et al., 2002; Dutilleul et al., 2003). Antioxidant enzymes protection systems in plants effectively scavenge ROS, and increased levels or activity of these enzymes can protect cell membranes from ROS damage. The three most important antioxidant enzymes that regulate the balance of ROS metabolism are superoxide dismutase (SOD), catalase (CAT), and peroxidase (POD; Wang et al., 2018).

The C-repeat-binding factor/dehydration-responsive element-binding factor 1 (CBF/DREB1) family is the most widely studied family of transcription factors associated with the low-temperature response of plants. Inducer of CBF expression 1 (ICE1) is a key transcriptional regulator of *CBF* genes, and together, they constitute the ICE1-CBF signaling pathway, which plays a critical role in the protection of plants against cold stress. CBF, belonging to the apetala2/ethylene response factor (AP2/ERF) family (Hannah, et al., 2006; Yamaguchi-Shinozaki, et al., 2006; Chinnusamy et al., 2010), binds to the promoter of the cold-related (*COR*) gene containing the dehydration-responsive element/C-repeat-binding element (DRE/CRT element) to induce the expression of the *COR* gene, which, in turn, affects the cold tolerance of plants. In the *Arabidopsis thaliana* genome, *CBF1 (DREB1B)*, *CBF2 (DREB1C)*, and *CBF3 (DREB1A)* have been successively identified as having high sequence similarity (Gilmour et al., 1998; Medina et al., 1999). Overexpression of *CBF1-3* was found to significantly increase the frost resistance of *A. thaliana* (Jaglo-Ottosen et al., 1998). Approximately 100 *COR* genes were detected as being expressed (Stockinger et al., 1997; Liu et al., 1998), and knockdown of *cbf1* and *cbf3* resulted in a 60% reduction in frost resistance in *A. thaliana* compared to the wild type (Novillo et al., 2007). To date, CBFs have been identified in *Brassica campestris*, *Triticum aestivum*, *Lycopersicon esculentum*, *Oryza sativa*, and *Zea mays* (Jaglo et al., 2001; Kasuga et al., 2004; Qin et al., 2004), and all these CBFs were found to exhibit cold-induced properties.

*Plumbago auriculata is a tropical semishrub native to South Africa, and its optimum growth temperature is 30–35°C. This species has been widely planted as a garden plant in various countries around the world because of its beautiful and rare blue corolla and is considered to be a model for studying the evolution of heterostyly (Ferrero et al., 2009). It has also been shown that the secondary metabolite plumbagin from *P. auriculata* directly inhibits the key PI3K/Akt/mTOR pathway in cancer (Hafeez et al., 2013; Li et al., 2014). In our previous study, we found that although *P. auriculata*, as a tropical plant, is very sensitive to low temperatures and that 15°C can cause growth arrest (Li et al., 2020), after cold acclimation in autumn, the plant was able to survive the winter in Chengdu, China, with a minimum temperature of −3°C. This ability to tolerate low temperatures was shown to be much better than that of conventional tropical plants (Zhang et al., 2004). However, the key pathways and related transcription factor regulatory processes in this plant associated with the response to low temperatures have not been elucidated. In this study, we measured the physiological indices of *P. auriculata* under cold stress, and transcriptome sequencing was performed. We found that cold stress at 4°C significantly reduced the photosynthetic efficiency of *P. auriculata* leaves and increased the activity of protective enzymes related to ROS scavenging. Transcriptome analysis identified a key transcription factor, *PaDREB1A*, that was highly expressed under cold stress in *P. auriculata*. The sequence of this transcription factor shared high homology with genes from other species. Upregulated expression of *COR* genes was observed in not only *P. auriculata* but also *A. thaliana* (ERD4, ERD7, and COR15A) after overexpression of *PaDREB1A*. At the same time, the level of ROS scavenging was enhanced, and the ability of plants to cope with cold stress was improved in transgenic *A. thaliana*. This is the first complete report on the discovery, isolation, identification, and functional study of a *P. auriculata* gene. We believe that in the context of rapid globalization, this study can serve as a typical case study for introducing tropical plants into regions with relatively low temperatures and provides an important theoretical basis for further molecular breeding to improve the tolerance of tropical plants to cold stress*.

## Materials and Methods

### Plant Material Growth Conditions and Cold Stress Treatment

One-year-old *P. auriculata* plants were planted in a greenhouse at Sichuan Agricultural University (Chengdu, China) and placed in a phytotron for 6 weeks prior to the experiment. Wild-type (WT) *A. thaliana* (Columbia ecotype, Col-0) and T3 generation transgenic lines were cultivated in the phytotron for 4 weeks. The cultivation conditions were as follows: photoperiod, 8 h/16 h (day/night); temperature, 25°C/20°C (day/night); light intensity 15,000 lx; and relative humidity, 70%.

For determination of the chlorophyll content, chlorophyll fluorescence parameters, protective enzyme activities, and MDA content, cold stress treatments were performed for 0, 3, 8, 24, and 72 h at 4°C in the dark. For RNA-seq and RT–qPCR, cold stress treatments were performed at 0, 1, 3, 8, and 24 h at 4°C in the dark. For morphological observations of WT and transgenic lines of *A. thaliana* under cold stress, freezing treatment was performed for 8 h at −6°C in the dark, and photography and recording of the results were performed after 24 h of recovery at room temperature.

### Determination of the Chlorophyll Content and Chlorophyll Fluorescence Parameters

The relative chlorophyll content of the leaves was measured directly using a SPAD-502 PLUS chlorophyll meter (Konica Minolta, Tokyo, Japan) *via* soil and plant analyzer development (SPAD) readings in the range of −9.9–199.9. Three plants were examined at each time point, and three points were randomly selected for each plant (Uddling et al., 2007).

Chlorophyll fluorescence parameters were measured using a Handy PEA plant efficiency analyzer (Hansatech, Norfolk, United Kingdom). Three plants were examined at each time point, and three points were randomly selected for each plant. Fv/Fm and PI abs were used to determine the photochemical efficiency of the leaves. The data points from 0 to 2 s were also recorded, and the fast chlorophyll fluorescence induction curve was plotted based on the data points (Rapacz, 2007).

### Assays of Protective Enzyme Activities and MDA Levels

These assays were carried out according to the manufacturer’s instructions. Leaves (0.1 g) were collected at each time point, with three biological replicates. Then, 0.1 ml of the extraction solution was added for milling in an ice bath, and the homogenate was centrifuged at 8000 × *g* and 4°C for 10 min. The SOD, POD, and CAT activities were measured with a BC0170 SOD assay kit (Solarbio, Beijing, China), POD BC0090 assay kit (Solarbio, Beijing, China), and BC0200 CAT assay kit (Solarbio, Beijing, China), respectively. The MDA content was measured with a BC0025 MDA assay kit (Solarbio, Beijing, China; Spitz and Oberley, 1989; Yin et al., 2018).

### RNA Preparation, RNA-Seq, and Differential Expression Analysis

One gram of leaves from each time point was collected and flash-frozen in liquid nitrogen with three biological replicates. Total leaf RNA was then extracted using a Pure Plant Kit (TIANGEN, Beijing, China), and RNA degradation and contamination were detected using 1% agarose gels. RNA quantification and qualification were performed using a Bioanalyzer 2,100 system (Agilent Technologies, CA, United States). The cDNA libraries were sequenced using the Illumina NovaSeq 6,000 System platform (Illumina, California, United States) *via* paired-end sequencing. Differential expression analysis of the two conditions/groups was performed using the DESeq R package (1.10.1). KOBAS (Xie et al., 2011) software was used to test the statistical enrichment of differentially expressed genes (DEGs) in Kyoto Encyclopedia of Genes and Genomes (KEGG) pathways.

### Cloning and Bioinformatic Analysis of PaDREB1A

Total RNA was extracted from *P. auriculata* leaves under cold stress, and cDNA was synthesized as a template by reverse transcription according to the instructions of the HiScript II 1st Strand cDNA Synthesis Kit (Vazyme, Nanjing, China). Primers (Supplementary Table S1) were designed with the full-length CDS obtained from RNA-seq using Primer 6 software for *PaDREB1A* gene cloning. The PCR products were detected by 1.0% agarose gel electrophoresis and subsequently recovered. The PCR products were transformed into *Escherichia coli* DH5α after construction of the pTOPO vector. The bacterial culture solution was tested again by PCR, and the screened monoclonal bacterial solution was verified by sequencing. Prediction of protein domains was performed using SMART.^1^ The amino acid sequence homology of PaDREB1A was examined using BLAST.^2^ Multiple sequence alignments were performed using DNAMAN 9.0, and ESPript 3.0^3^ was used for mapping. MEGA 7.0 was used for phylogenetic tree mapping.

### Vector Construction and Generation of Transgenic Lines

To produce the *35S::PaDREB1A* lines, the target gene was amplified using appropriate primers (Supplementary Table S1). The *pCAMBIA1302-PaDREB1A-EGFP* overexpression vector (Supplementary Figure S1) was constructed using *Nco*I enzyme cleavage of the vector and homologous recombination. Monoclonal bacterial solution was screened by PCR. After sequencing and verification, the plasmids were extracted and transformed into *Agrobacterium tumefaciens* GV3101 receptor cells. After incubation at 28°C for 36 h, monoclonal bacterial solution was selected for PCR identification. *A. thaliana* plants were transformed using the floral-dip transformation method. The transformed *A. thaliana* plants were cultured until seeds were harvested. The harvested seeds were sterilized and uniformly dispersed in 1/2 MS medium (with the addition of hygromycin) and cultured until the 4-leaf stage. Plants that were screened as being positive for resistance were transferred to soil for growth. The vector was also tested using universal primers (Supplementary Table S1) to identify transgenic T0 generation plants (Qiu et al., 2020).

### RT–qPCR Validation

The RT–qPCR assay was divided into three parts: (1) five genes were selected for RT–qPCR to verify the reliability of RNA-seq, (2) the expression of *PaDREB1A* in 16 transgenic lines was examined, and (3) four related genes were selected to verify the regulatory effects of *PaDREB1A* on downstream *CORs* and oxidative stress-related protective enzymes (*ERD4, ERD7, COR15A, CAT1*). The TUREscript 1st Strand cDNA Synthesis Kit (Nobelab Biotechnologies, Beijing, China) was used to synthesize cDNA following the manufacturer’s instructions. Primers were designed using Premier 6. The primer sets for the target and reference genes are listed in Supplementary Table S1. RT–qPCR was performed with qTOWER 2.0/2.2 Quantitative Real-Time PCR Thermal Cyclers (Analytik Jena, Jena, Germany). Relative gene expression levels were calculated automatically using qPCRsoft3.2 software.

### Data Analysis

Data were processed and analyzed using Microsoft Excel 365 and IBM SPSS Statistics 26. One-way ANOVA was used to determine significant differences. For RNA-seq, differential expression analysis was performed with the DESeq R package (1.10.1). Unigenes with a false discovery rate (fDR) < 0.01 and fold change (fC) ≥ 2 identified by DESeq analysis were considered DEGs.

## Results

### Chlorophyll Content and Chlorophyll Fluorescence Parameters of *P. auriculata* Under Cold Stress

**Figure 1:**
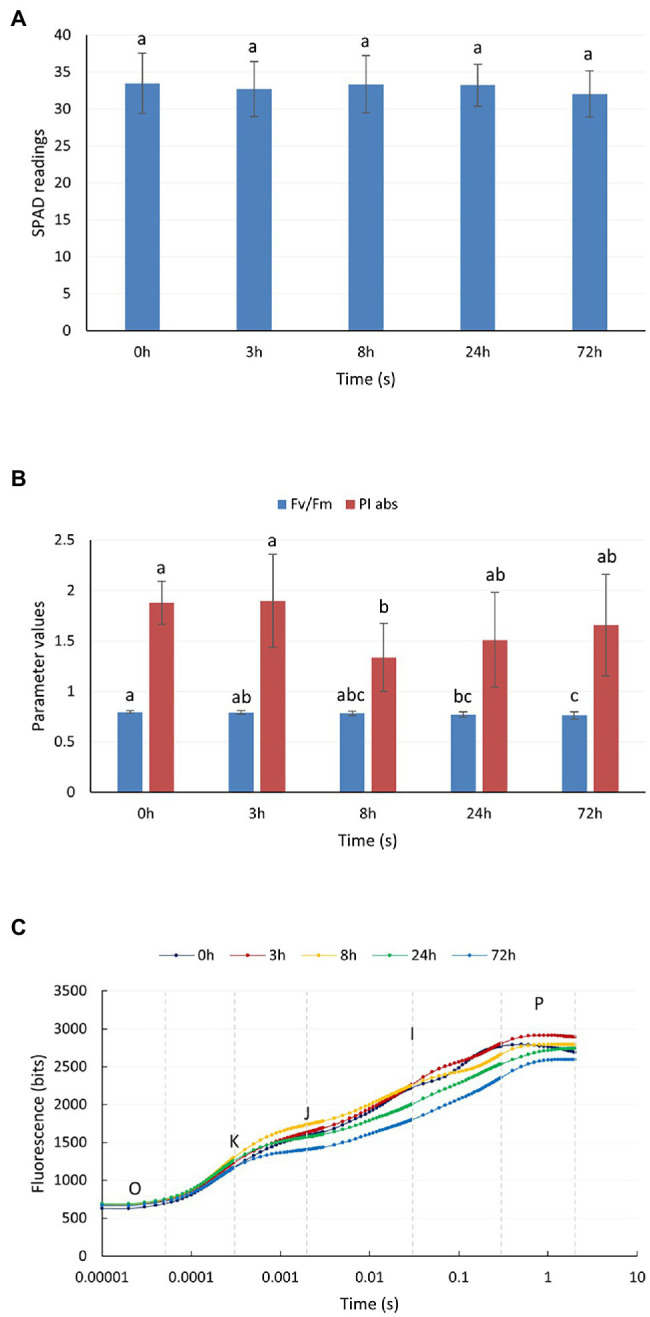
Chlorophyll content and chlorophyll fluorescence parameters of *P. auriculata* after different cold stress treatment times. **(A)** Relative chlorophyll content. **(B)** Chlorophyll fluorescence parameters. **(C)** Fast chlorophyll fluorescence induction curve. O, K, J, I, and P are the characteristic sites of the chlorophyll fluorescence induction curve. Means with the same letters are non-significantly different at different times.

Figure 1A shows the changes in the relative chlorophyll content of *P. auriculata* after the plants were subjected to cold stress at 4°C. The relative chlorophyll content of the leaves did not vary significantly from 0 to 72 h. Two parameters, namely, PI abs and Fv/Fm, could indicate the response of plant photochemical efficiency to stress, especially PI abs, which could respond to very small changes in the environment. For PI abs (Figure 1B), a significant decrease started to appear after 8 h of cold stress at 4°C, but the value started to gradually rebound after 24 h. The Fv/Fm value started to decrease at 24 h. This observation indicated that cold stress at 4°C caused some degree of damage to the leaves of *P. auriculata*, but this damage was mild, and the leaves gradually recovered during plant adaptation. Figure 1C shows the fast chlorophyll fluorescence induction curve after different durations of cold stress. The curve showed a significant decrease at 24 h and a more pronounced decrease at 72 h. In particular, two characteristic points, J and I, showed a downward shift, indicating a decrease in photochemical efficiency at a low temperature.

### Protective Enzyme Activities and MDA Content of *P. auriculata* Under Cold Stress

**Figure 2:**
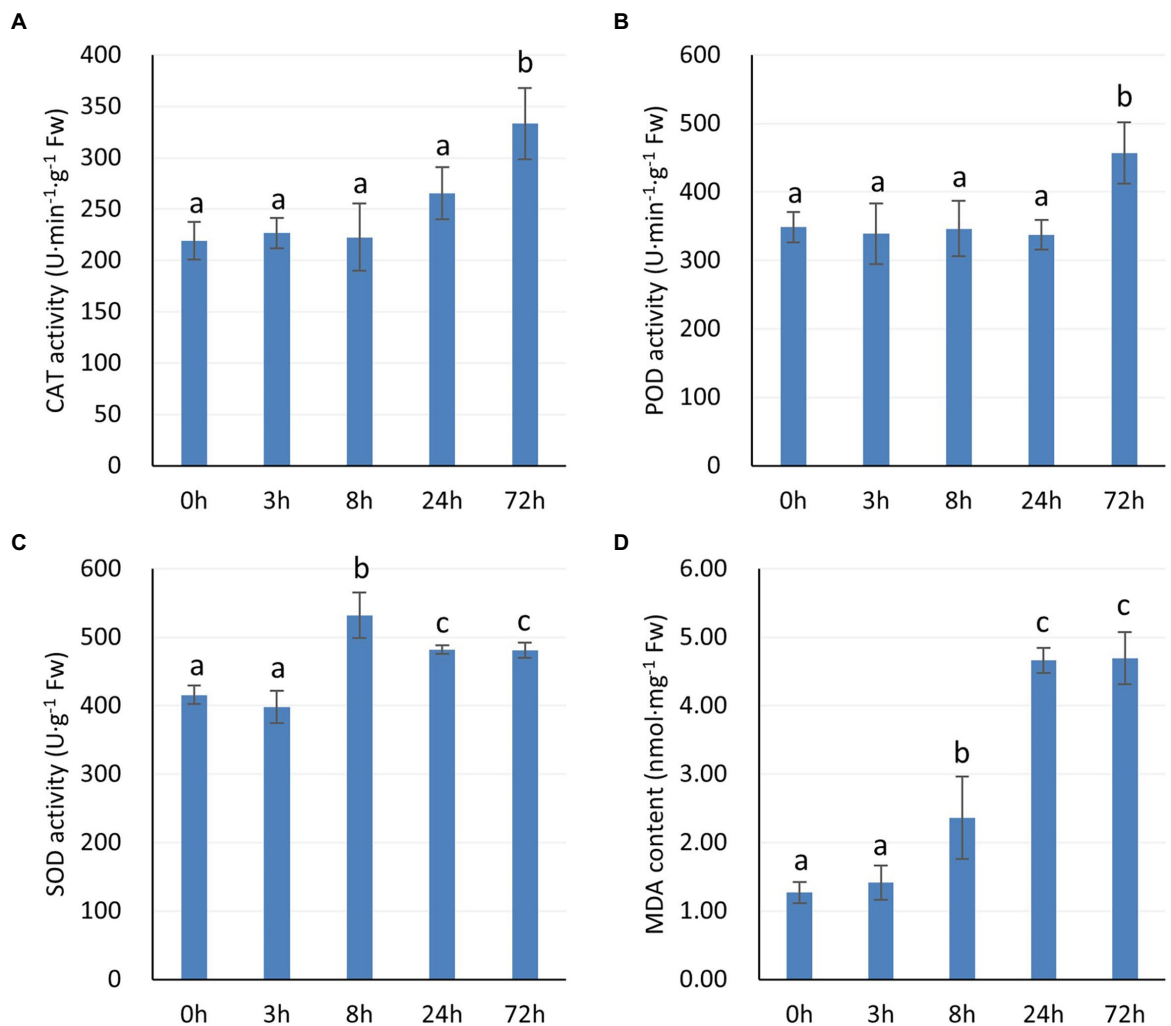
Activities of protective enzymes and MDA content in *P. auriculata* after different cold stress treatment times. **(A)** CAT activity. **(B)** POD activity. **(C)** SOD activity. **(D)** MDA content. Means with the same letters are non-significantly different at different times.

Relatively high antioxidant enzyme activities may reduce the oxidative damage suffered by plant leaves under stress. Figure 2 shows the protective enzyme activity and MDA content of *P. auriculata* at different times under 4°C cold stress. The CAT and POD activities showed a significant increase at 72 h (Figures 2A,B). However, the SOD activity peaked at 8 h, showed a decrease at 24 h, and then maintained a relatively stable value (Figure 2C). The MDA content started to show a significant increase at 8 h, peaked at 24 h, and then stopped increasing (Figure 2D). This observation indicates that the accumulation of ROS after cold stress was significant, but the value stabilized after 24 h. Thus, after 24 h, these three protective enzymes may play an important role in the cold tolerance of *P. auriculata*.

### RNA-Seq and Assembly and Differential Expression Analysis

Transcriptome sequencing of 15 samples (three biological replicates) was conducted, and a total of 118.16 Gb of clean data was obtained. The amount of clean data for each sample reached 6.18 Gb, with a Q30 base percentage of 93.79% and above. A total of 40,726 unigenes were obtained after assembly. Among them, there were 14,037 unigenes with lengths above 1 kb. The unigenes were functionally annotated with the NR (Deng et al., 2006), Swiss-Prot (Apweiler et al., 2004), KEGG (Kanehisa et al., 2004), COG (Tatusov et al., 2000), KOG (Koonin et al., 2004), GO (Ashburner et al., 2000), and Pfam (finn et al., 2014) databases, obtaining a total of 17,554 unigene annotations. Selected unigenes of interest were subjected to RT–qPCR analysis, the reliability of the RNA-seq results was verified, and the expression trends of all the selected genes were found to be consistent with the RNA-seq results (Supplementary Figure S2).

Four different cold stress treatment times were compared with 0 h as the control, and the comparison combinations were as follows: S1 (0 h vs. 1 h), S2 (0 h vs. 3 h), S3 (0 h vs. 8 h), and S4 (0 h vs. 24 h). Analysis of DEGs and related pathways was performed. The number of DEGs increased with increasing cold stress treatment time. For S1, there were only 53 DEGs, but for S4, 2,578 DEGs were obtained. Notably, the differences in the number of upregulated and downregulated genes for S1, S2, and S3 were not significant. However, the number of upregulated genes (1524) was much higher than the number of downregulated genes (1054) for S4 (Supplementary Figure S3). This may be related to the positive response of related genes in the process of plant resistance to cold stress.

### KEGG Pathway Identification

KEGG enrichment factor analysis of DEGs was performed for the four comparison combinations. We collated the pathways with a Q-value ≤0.6 (Supplementary Figure S4). For S1, which represented the initial stage of cold stress response, only a small number of DEGs were annotated. Zeatin biosynthesis (ko00908) was found to be a downregulated pathway, which implies that zeatin may be involved in the stress signaling response at an early stage. For S2, two pathways of lipid metabolism, namely, linoleic acid metabolism (ko00591) and alpha-linolenic acid metabolism (ko00592), were upregulated. In particular, one of the catabolic products of linolenic acid is jasmonic acid (JA), an important growth regulator involved in the cold stress response. At the same time, upregulation of the secondary metabolite synthesis pathway tropane, piperidine, and pyridine alkaloid biosynthesis (ko00960) was also observed. This result may be related to the plant defense. ABC transporters (ko02010) and pentose and glucuronate interconversions (ko00040) were downregulated, which may indicate a slowdown in plant metabolism under cold stress. For S3, plant hormone signal transduction (ko04075) was upregulated, confirming our previous hypothesis. Further analysis of this pathway revealed that all three genes associated with JA signaling, namely, *JAR1, JAZ*, and *MYC2* (Supplementary Figure S5), were upregulated. Brassinosteroid biosynthesis (ko00905) and photosynthesis–antenna proteins (ko00196) were downregulated in S3 and S4. The downregulation of photosynthesis–antenna proteins was consistent with the chlorophyll fluorescence results, explaining the reduction in photochemical efficiency of the plants after cold stress.

### Transcription Factor Prediction and CBF-Mediated Pathway Annotation

**Figure 3:**
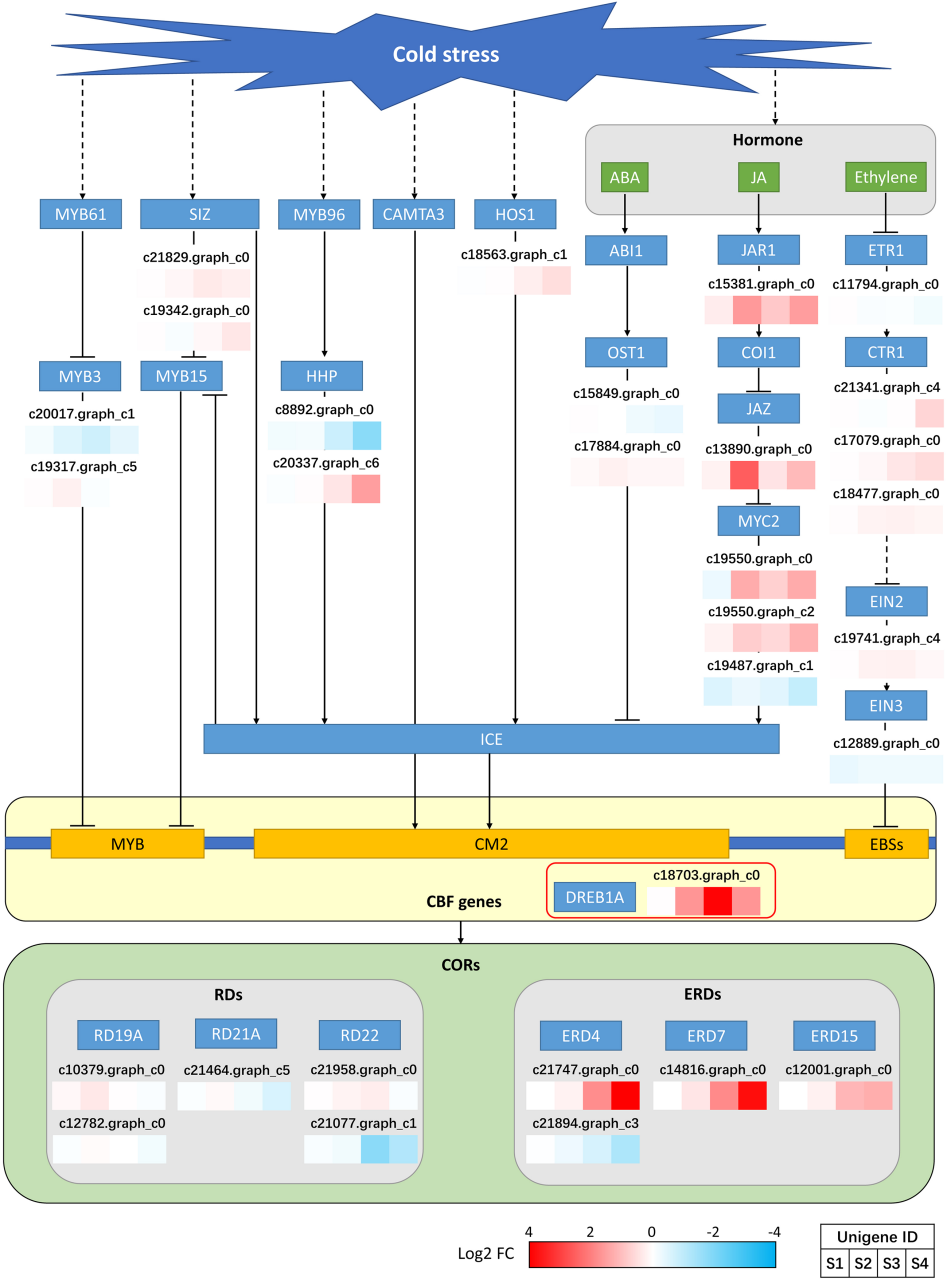
Expression of CBF-mediated upstream and downstream genes in *P. auriculata* after different cold stress treatment times.

The transcription factors were predicted, and the results of the top 20 predictions are shown in Supplementary Figure S6. The three most abundant transcription factors belonged to the AP2/ERF-ERF (65), bHLH (55), and NAC (55) families. In addition, other transcription factors, such as those of the MYB (39), WRKY (35), and bZIP (21) families, which may be involved in the cold stress response, were also predicted. We focused on the expression of CBF/DREB1 transcription factors that are important for the regulation of cold stress in plants. Finally, c18703.graph_c0 was targeted. This unigene was annotated as *DREB1A* and started to be upregulated at S2, with expression peaking at S3 and starting to decline at S4 (Figure 3). The *DREB1A* expression pattern was fully consistent with that of CBF-mediated cold stress response genes (Su et al., 2010). Annotation analysis was conducted for several upstream genes and hormone signaling pathways that may be involved in CBF regulation. However, most of the genes were not significantly differentially expressed (Figure 3), but the pathways involved in JA regulation appeared to be differentially expressed. MYC2, an upstream positive regulator of *ICE*, had two unigenes that were significantly upregulated in S2 and S4 and one unigene that was significantly downregulated in S4. JAR1, located upstream of *MYC2*, is a positive regulator of *MYC2*, and JAZ is a negative regulator. However, both of these genes were found to be significantly upregulated. This is a complex set of mixed regulators, and we hypothesize that other signaling pathways are involved, but further clarification is required. However, based on the expression of *MYC2*, we hypothesize that JA is most likely involved in the response to cold stress in *P. auriculata* and positively regulates the expression of *CBFs*. In addition, we annotated the downstream *CORs* that may be regulated by *DREB1A*. The annotated *CORs* were divided into two main categories: *RDs* and *ERDs*. There was no significant difference in the expression of *RDs*, but the expression of *ERD4* and *ERD7* was significantly different at different treatment times, with expression gradually increasing, peaking at S4 and lagging behind that in DREB1A (Figure 3). The expression pattern of these two genes was fully consistent with the expression pattern of *CORs* in response to cold stress (Shi et al., 2018). Therefore, we suggest that *DREB1A* may be a key transcription factor involved in the regulation of cold stress in *P. auriculata* and regulates the two *CORs* downstream to promote cold resistance.

### Isolation and Characterization of PaDREB1A

**Figure 4:**
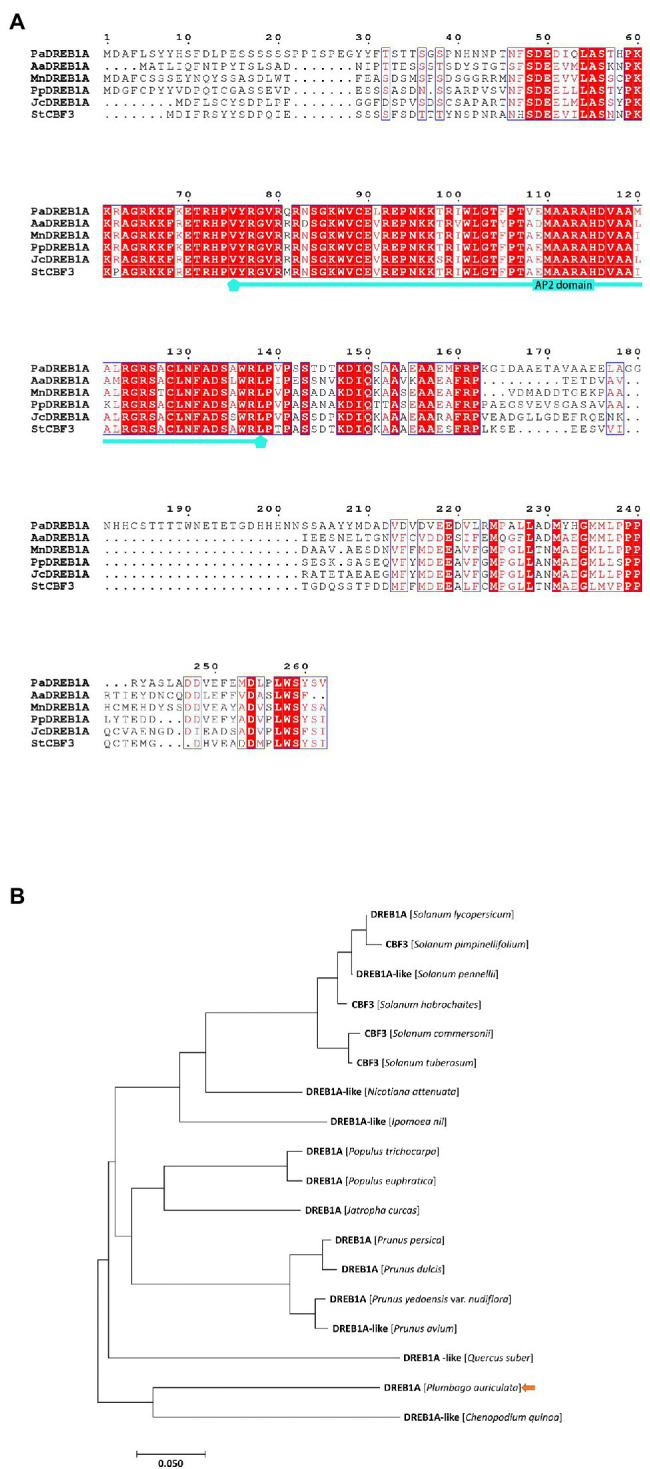
Sequence alignment and phylogenetic analysis of *PaDREB1A*. **(A)** Multiple sequence alignment of *PaDREB1A*. **(B)** Phylogenetic tree constructed using the neighbor-joining method. *PaDREB1A* is indicated by an arrow.

Total RNA was extracted from *P. auriculata* leaves after 8 h of cold stress treatment and reverse transcribed to cDNA as a template. The CDS of the *DREB1A* gene obtained from RNA-seq was used to design multiple primer pairs for PCR amplification. The gel electrophoresis map showed a bright band at 750 bp. The band was determined to match the size of the target gene 789 bp (Supplementary Figure S7) and was named *PaDREB1A* (Accession number: MZ686430). *PaDREB1A* encodes 262 amino acids and has a predicted molecular mass of 29.1 kDa. SMART analysis showed that it contains a typical AP2 domain. Multiple sequence alignment with the other five homologous DREB1A proteins showed high conservation of its AP2 domain compared with those from other plant species (Figure 4A). Notably, the conserved amino acid residue in the AP2 domain was changed from V to L at position 91 and from A to V at position 108, unlike in other plants. Phylogenetic analysis showed that the closest PaDREB1A relative was the *Chenopodium quinoa* DREB1A-like protein (Figure 4B).

### Overexpression of PaDREB1A Enhanced Cold Tolerance

**Figure 5:**
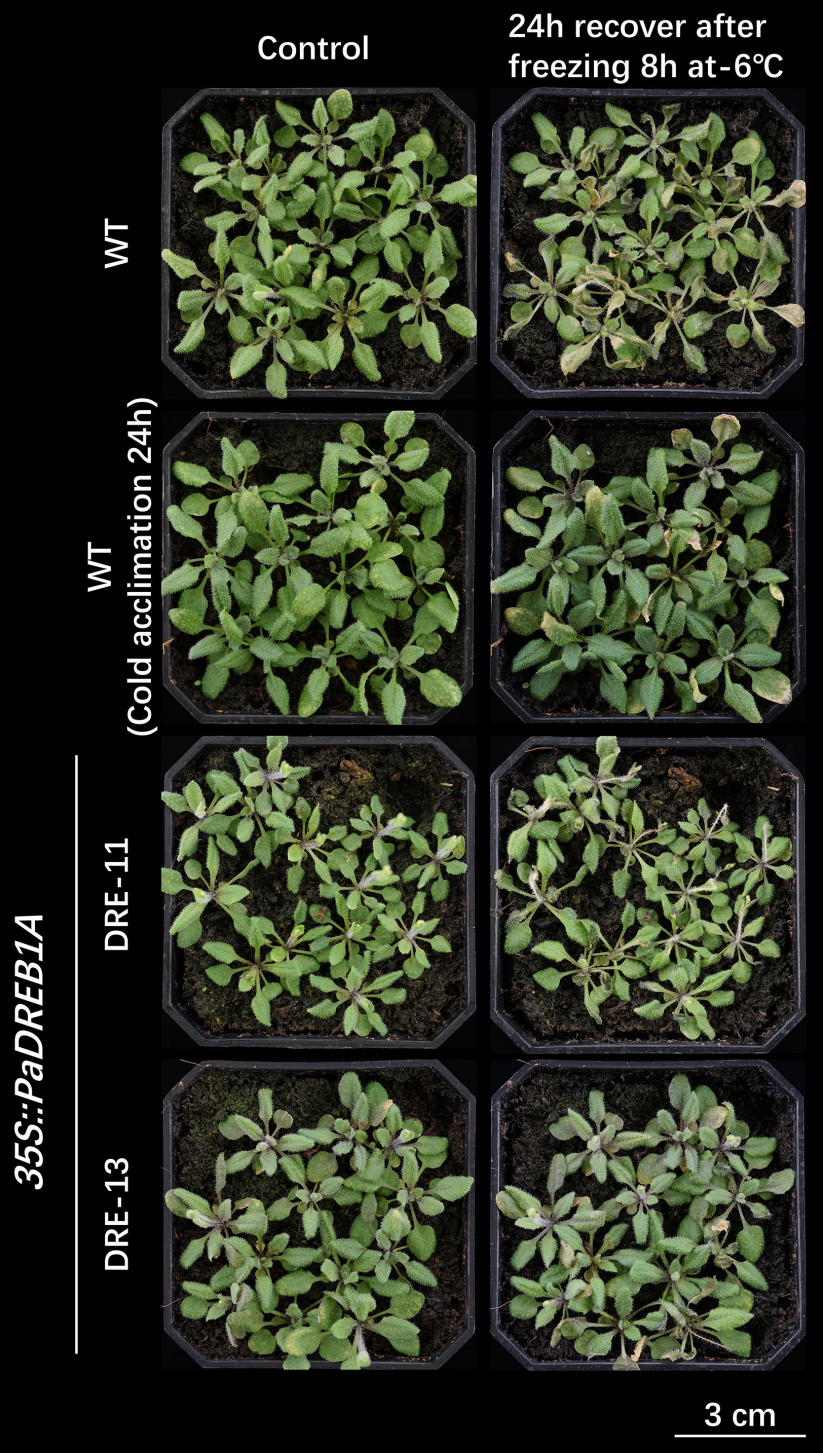
Cold stress analysis of *PaDREB1A* transgenic lines and WT plants.

To investigate the role of *PaDREB1A* in cold stress, the *pCAMBIA1302-PaDREB1A-EGFP* vector was constructed through *A. tumefaciens*-mediated transformation of *A. thaliana*. T0 generation plants were screened for resistance to hygromycin, and 22 strains were obtained. A total of 16 strains showed stable expression after a universal primer assay of the vector (Supplementary Figure S8A). After RT–qPCR, two positive transformants, namely, DRE-11 and DRE-13, were selected as the two most highly expressed strains (Supplementary Figure S8B). These transformants were again screened for hygromycin resistance and used as the third generation for the subsequent experiment. Adult plants grown in soil for 4 weeks were used for the cold stress treatment. WT plants showed symptoms of extensive leaf wilting after 8 h of freezing treatment at −6°C. WT plants (after 24 h of cold acclimation at 4°C) were significantly more resistant to freezing, with only some leaf edges wilted. Both transgenic strains showed significantly less leaf damage after freezing treatment than the WT plants without cold domestication, especially DRE-13, which exhibited frost resistance close to that of the plants after cold domestication (Figure 5). After a longer recovery period of 72 h, all strains had withered leaves, but showed similar results as before. WT plants without cold acclimation had the most withered leaves. The damage of DRE-13 was similar to that of WT plants after cold acclimation, with less damage (Supplementary Figure S9).

### Overexpression of PaDREB1A Protected the Photosynthetic System Under Cold Stress

**Figure 6:**
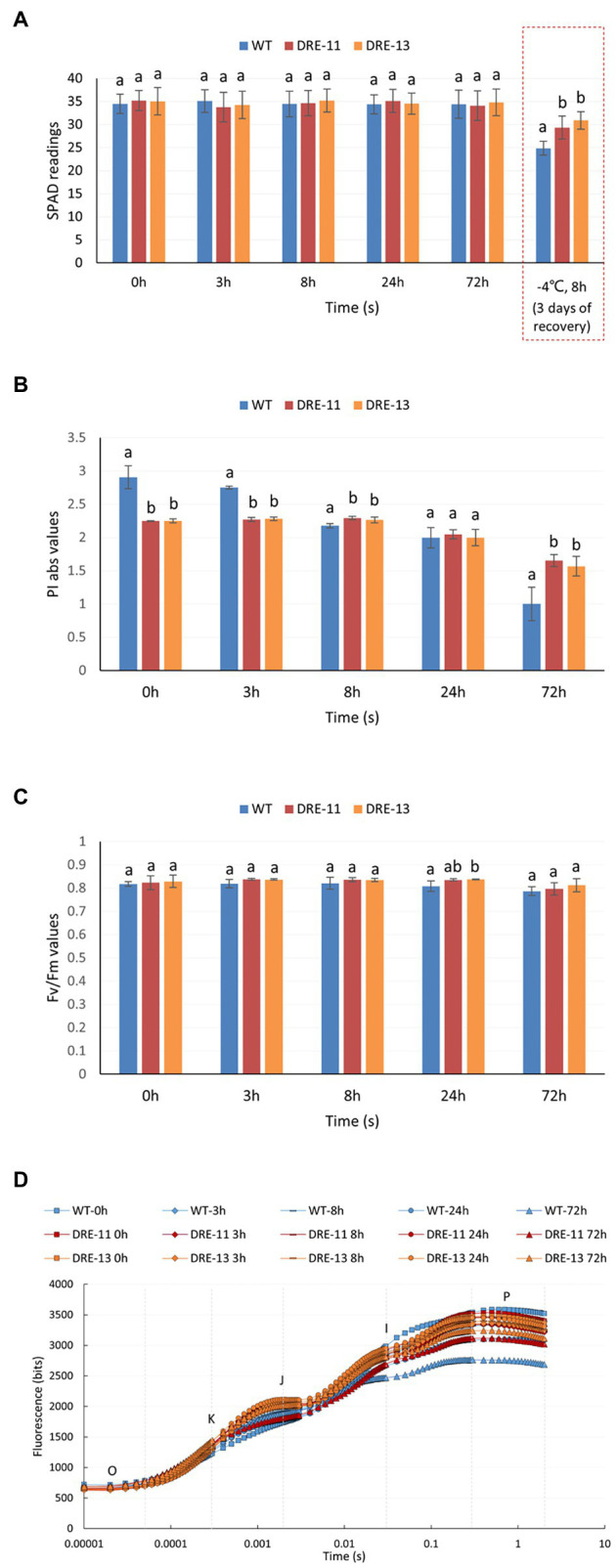
Chlorophyll content and chlorophyll fluorescence parameters of *PaDREB1A* transgenic lines and WT plants after different cold stress treatment times. **(A)** SPAD readings. The dotted box shows an additional set of ice stress tests. **(B)** PI abs values and **(C)** Fv/Fm values. Means with the same letters are non-significantly different for different transgenic lines and WT plants. **(D)** Fast chlorophyll fluorescence induction curve. O, K, J, I, and P are the characteristic sites.

As shown in Figure 6A, similar to the results reported in section “Chlorophyll Content and Chlorophyll Fluorescence Parameters of *P. auriculata* Under Cold Stress,” neither WT nor the two transgenic strains showed a significant difference in relative chlorophyll content after 72 h of cold stress at 4°C. We hypothesized that a low temperature of 4°C would not have a significant effect on the chlorophyll content of the plants in a short period of time. Therefore, freezing treatment was conducted based on the observations in section “Overexpression of PaDREB1A Enhanced Cold Tolerance.” We subjected the three plants to freezing treatment at −4°C for 8 h, allowed them to recover at room temperature for 3 days, and then measured the chlorophyll content in the leaves, and the data appeared to be significantly different. The WT plants were significantly more severely affected, with a significantly lower chlorophyll content (24.87 ± 1.51) than that of the two transgenic strains (29.33 ± 2.46; 30.88 ± 1.91).

The chlorophyll fluorescence parameters more sensitively reflected the impact of stress on the plant photochemical system than the chlorophyll content. WT plants had significantly higher values of PI abs than the two transgenic strains before cold stress (Figure 6B). This indicated that the transgenic strains may have a lower maximum photochemical efficiency than the WT plants. However, WT plants were significantly more sensitive to low temperature; their PI abs values were already on par with those of the two transgenic strains after 24 h of treatment, while they were already much lower than those of the two transgenic strains at 72 h. In contrast, the PI abs values of the two transgenic strains DRE-11 and DRE-13 changed moderately. Neither WT nor transgenic plants showed significant differences in Fv/Fm (Figure 6C). Fv/Fm is more stable than the value of *P*, but it is also less sensitive. No significant change in Fv/Fm was observed in this study, indicating that the plant may have suffered from cold stress at an early stage. The chlorophyll fluorescence induction curves showed similar results to those shown in *P. auriculata* (Figure 1C). WT plants showed different degrees of decrease in the curve at all stress time points, especially at 72 h, when the I point had almost completely disappeared. Again, this represents a significant impact on photochemical efficiency. In contrast, the two transgenic strains, especially DRE-13, were less strongly affected than the WT plants, as the curves started to show only a very slight decrease at 72 h (Figure 6D).

### Overexpression of PaDREB1A Increased the ROS Scavenging Capacity

**Figure 7:**
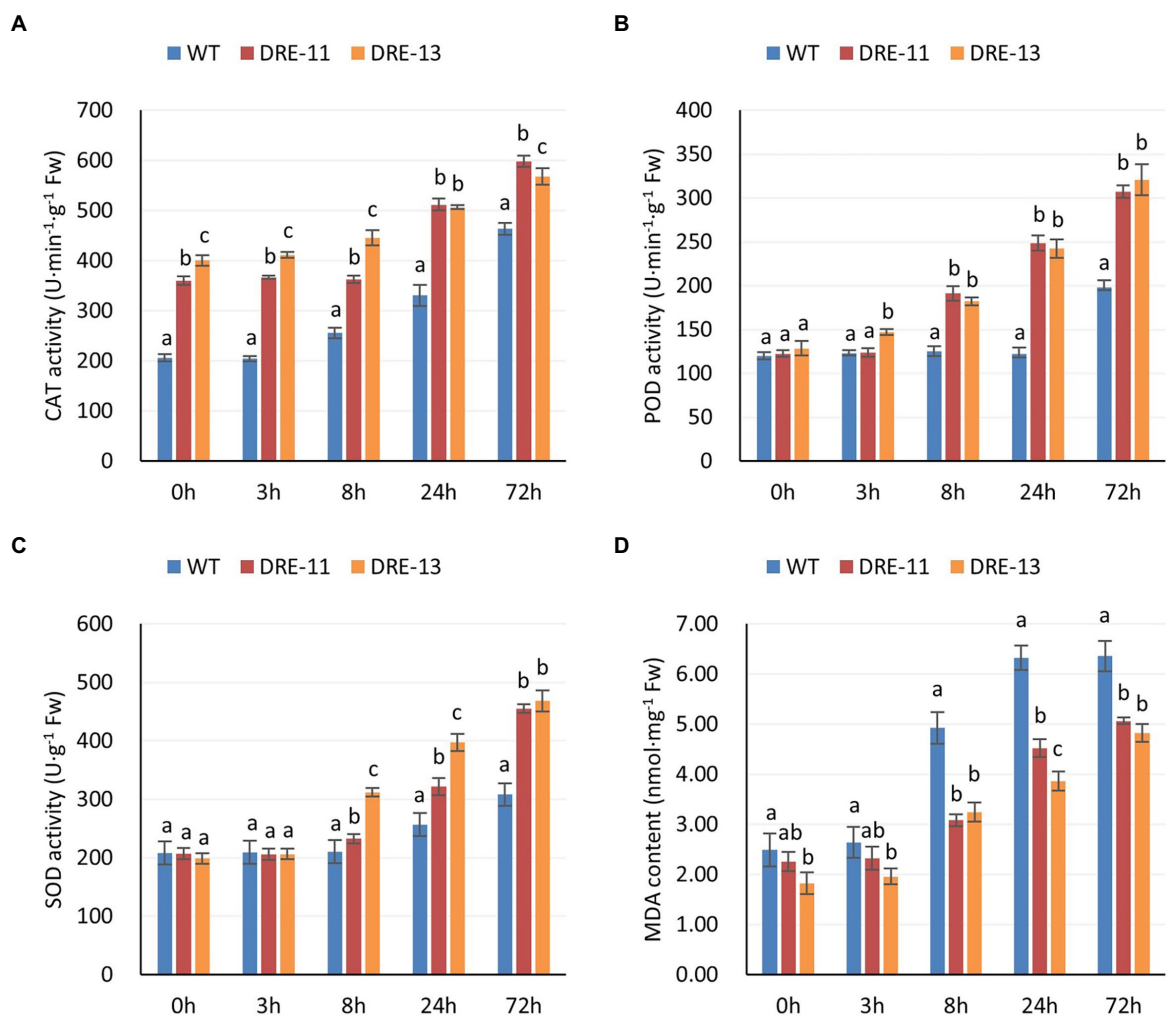
Activities of protective enzymes and MDA content of *PaDREB1A* transgenic lines and WT plants after different cold stress treatment times. **(A)** CAT activity. **(B)** POD activity. **(C)** SOD activity. **(D)** MDA content. Means with the same letters are non-significantly different for different transgenic lines and WT plants.

CAT, SOD, and POD all play important roles in the scavenging of ROS in plants. CBFs can enhance the expression of downstream *CORs* and thus indirectly enhance the ROS scavenging mechanism in plants. As shown in Figure 7, both transgenic strains showed higher CAT activity than the WT plants even when they were not under cold stress, and the levels remained significantly higher at subsequent times (Figure 7A). In contrast, the SOD and POD activities did not show a significant difference at the beginning of the stress period. However, their activities soon significantly exceeded those of the WT plants with time (Figures 7B,C). The MDA content is an indicator of the extent of lipid peroxidation in plants. As shown in Figure 7D, the MDA content of WT plants was significantly higher than that of the two transgenic strains, and this difference was more pronounced in the 8 h samples. These results indicate that overexpression of *PaDREB1A* enhanced the scavenging ability of ROS in the plants and reduced the oxidative damage of plants under cold stress.

### Overexpression of PaDREB1A Upregulated the Expression Levels of CORs and CAT1

**Figure 8:**
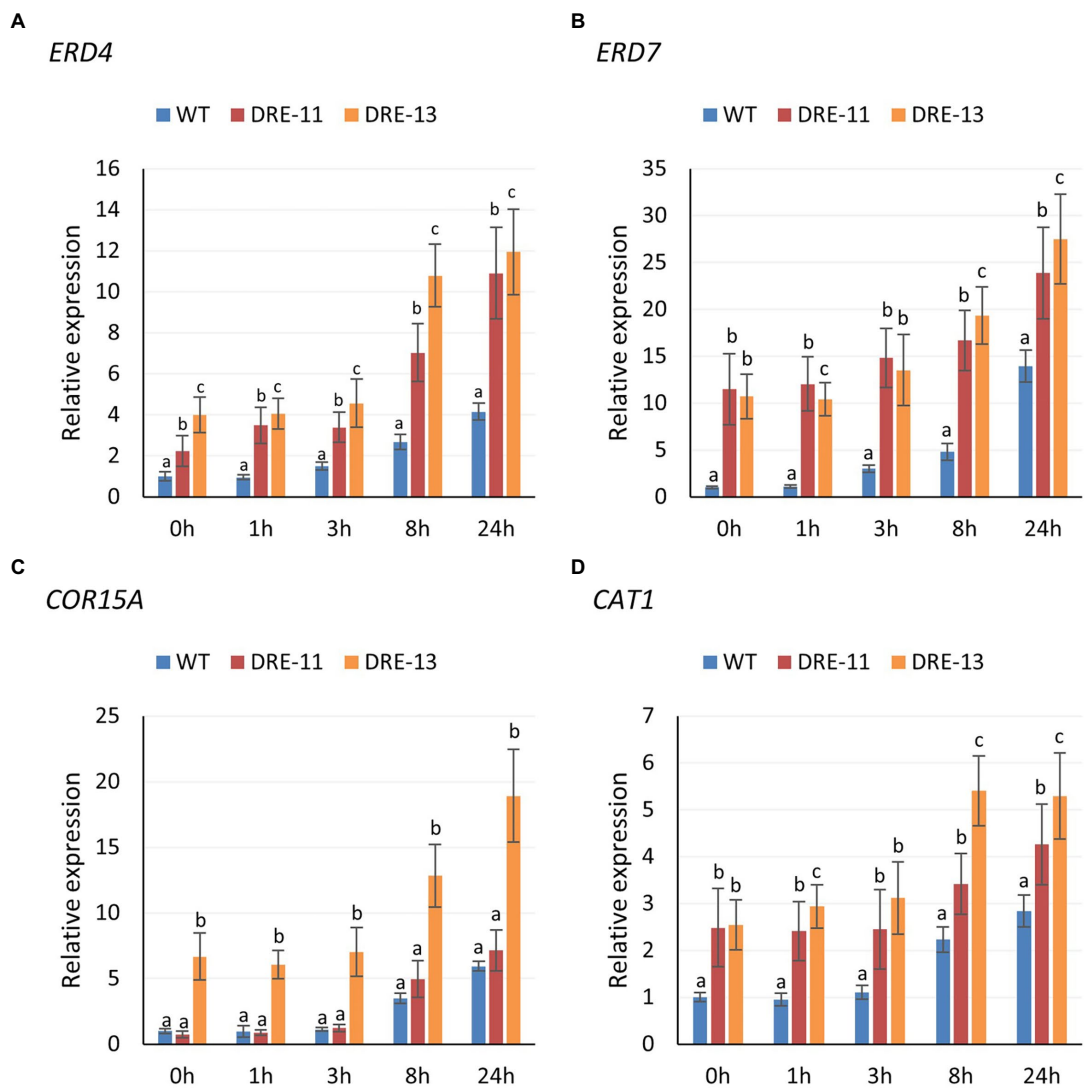
Expression levels of *CORs* (**A**, *ERD4*; **B**, *ERD7*; **C**, *COR15A*) and **(D)** *CAT1* of *PaDREB1A* transgenic lines and WT plants after different cold stress treatment times. Means with the same letters are non-significantly different for different transgenic lines and WT plants.

Since we found that two *COR* genes, namely, *ERD4* and *ERD7*, were upregulated with *PaDREB1A* overexpression in the transcriptome data, we hypothesized that *PaDREB1A* could similarly upregulate *COR* gene expression downstream in *A. thaliana*. We selected three *CORs*, including the previously mentioned *ERD4, ERD7*, and *COR15A*, for RT–qPCR assays. We also determined the change in *CAT1* expression due to the significant enhancement of ROS scavenging ability. The results revealed that the expression of *ERD4* and *ERD7* was significantly upregulated in transgenic plants compared with WT plants, as expected from our transcriptomic results (Figures 8A,B). However, for *COR15A*, only DRE-13 showed significantly upregulated expression, while the expression level in DRE-11 was close to that in WT plants (Figure 8C). In addition, the *CAT1* expression levels in both transgenic plants were also significantly higher than those in WT plants. This again demonstrated that overexpression of *PaDREB1A* could enhance the ROS scavenging level in the plants (Figure 8D). Although the expression of *ERD4, ERD7*, and *CAT1* was enhanced in both transgenic strains, the expression level in DRE-13 was significantly stronger than that in DRE-11 at the late stage of stress. This result may be one of the reasons for the enhanced cold stress tolerance of the transgenic plants.

## Discussion

Among all physiological processes in plants, photosynthesis is the most sensitive to the effects of low temperature. Cold stress can disrupt the chloroplast structure, decrease the chlorophyll content, and impair the electron transport function in plants. SPAD values have been widely used to assess the decrease in chlorophyll content caused by various abiotic stresses on plants. In pepper (*Capsicum frutescens*), leaves subjected to cold stress exhibited a significant decrease in SPAD values (Odhiambo et al., 2018). Similar findings have been obtained in other plants, and the extent of this decrease depends on the species itself (Miedema, 1982). In this study, we found that the SPAD values of *P. auriculata* under cold stress remained almost unchanged for 72 h. This result indicates that the chloroplasts of *P. auriculata* leaves were not structurally damaged by cold stress treatment at 4°C for a short period of time, and the chlorophyll level was maintained. Chlorophyll fluorescence reflects damage to PSII in plant leaves exposed to abiotic stress, and the data can be obtained quickly and efficiently without damaging the leaves. The maximum photochemical efficiency of PSII (Fv/Fm) is one of the most important chlorophyll fluorescence parameters. PI abs is the most sensitive of all chlorophyll fluorescence parameters, responding rapidly to subtle changes (Baker and Rosenqvist, 2004; Baker, 2008). These parameters have been widely used in response to cold stress in plants, such as soybean, tomato, and maize (Guy et al., 1997; Skrudlik et al., 2000; Cao et al., 2015). For *P. auriculata*, cold stress treatment at 4°C for 72 h caused a decrease in the photochemical efficiency of *P. auriculata* leaves but did not produce irreversible destructive damage. Many studies have shown that cold stress can cause ROS accumulation in plants, trigger membrane lipid oxidation, and activate elevated protective enzyme activities (Faize et al., 2011; Bhattacharjee, 2013; Diaz-Vivancos et al., 2013). Similar results were obtained in this study, where the CAT, SOD, and POD activities in the leaves of *P. auriculata* gradually increased with the duration of cold stress. However, we found that the CAT and POD activities did not peak. That is, the activities of these protective enzymes may be further increased if the stress time is further prolonged. However, the MDA content did not increase further between 24 h and 72 h for any plants, which indicates that the ROS content in the leaves of *P. auriculata* was well controlled and that further membrane lipid oxidation did not occur.

Several results from the current study show that cold stress can upregulate the tropane, piperidine, and pyridine alkaloid biosynthesis pathways. Song et al. (2020) found that this pathway in Tartary buckwheat (*Fagopyrum tataricum*) is also upregulated under cold stress. Accumulation of these substances may be induced at low temperature and promote cold tolerance. Similar trends have also been observed in maize and wild banana (*Musa itinerans*; Liu et al., 2018; Li et al., 2019). In this study, we also found that the tropane, piperidine, and pyridine alkaloid biosynthesis pathways were significantly upregulated in *P. auriculata* after cold stress, and we thus hypothesize that *P. auriculata* may have a similar mechanism to resist cold stress. In addition, we noted that another pathway, pentose and glucuronate interconversions, was also significantly upregulated under cold stress. This phenomenon has also been found in *Cardiocrinum cathayanum* and may significantly improve its resistance to cold (Wang et al., 2020). In fact, upregulation of the pentose and glucuronate interconversions pathway has been found in a variety of plants, and this upregulation may be an important mechanism leading to plant cold tolerance (Gong et al., 2018; Zhang et al., 2020). JAs are composed of JA and its derivatives, such as methyl jasmonate (MeJA) and jasmonoyl–isoleucine, which play a role in growth and development as well as in biotic and abiotic stress responses (Ding et al., 2021). It has been widely demonstrated that cold stress can induce endogenous JA accumulation and act as an upstream signal for ICE-CBF to positively regulate cold tolerance in *A. thaliana* (Hu et al., 2013). As found in tomato, cold stress was able to induce JA production, and JA signaling further activated the CBF pathway to increase cold tolerance (Wang et al., 2016). MYC2 is a major regulator of the JA signaling pathway and directly interacts with ICE1 to jointly activate cold tolerance in banana (Zhao et al., 2013). MdMYC2 can activate the G-BOX element in the *MdCBF1* promoter of apple (*Malus* × *domestica*) to improve freezing tolerance. MdJAZ1/4 can bind to *MdMYC2* and act as a negative regulator to reduce the expression of *MdMYC2* and downstream *CBFs* (Wang et al., 2019). In this study, transcriptome analysis of *P. auriculata* under cold stress repeatedly upregulated the pathways involved in JA synthesis and signal transduction (linoleic acid metabolism, ko00591; plant hormone signal transduction, ko04075), and it was further observed that *JAR1, JAZ*, and *MYC2*, genes directly related to JA signaling, were upregulated. The upregulated genes included *JAR*, which is located most far upstream of and positively regulates the *CBF* gene; JAZ, which is located downstream and is a negative regulator; and MYC2, which is located most far downstream of and positive regulates *ICE* and *CBF*. According to the results of transcriptome analysis, MYC2 expression in *P. auriculata* was upregulated in S2. We hypothesize that endogenous JA may play a positive regulatory role in the CBF signaling pathway. However, this regulation was complex, and multiple unigenes were annotated to the same genetic locus. Moreover, inconsistent expression was observed, and both the positive regulator *JAR* and the negative regulator *JAZ* appeared to be upregulated in S2. Thus, it may be the case that there are other signaling pathways involved in regulation. Our findings suggest that JA may be an important signaling substance for the upstream regulation of CBFs in *P. auriculata*, but the mode of regulation of its signaling pathway is not clear from the present information and requires further study.

Cold stress leads to a decrease in photosynthesis, resulting in an increase in the total amount of excess excitation energy (Payton et al., 2001). If the excess light energy is not dissipated in a timely manner, the photosynthetic system can be damaged even under low light (Demmig-Adams and Adams, 1992), causing photoinhibition (Allen and Ort, 2001). In this study, all cold stress experiments were carried out under dark conditions to avoid interference from further damage to the photosynthetic system by photoinhibition. The decrease in photosynthesis in *P. auriculata* was partly caused by the decrease in enzyme activity at low temperatures and the disruption of the membrane system by ROS. Transcriptome analysis was performed, and the results showed significant downregulation of the photosynthesis–antenna protein (ko00196) pathway in both S3 and S4. The downregulated genes were involved mainly in the light-harvesting chlorophyll protein complex (LHC) system of *Lhca4, Lhcb1*, and *Lhcb3* (Supplementary Figure S10). We believe that this is a rational response for plants to autonomously regulate the reduction of light energy uptake to allow plants to protect themselves from cold stress, which can mitigate photoinhibition and cause further damage.

To date, CBF1 (DREB1B), CBF2 (DREB1C), and CBF3 (DREB1A) have been identified successively in *A. thaliana* (Gilmour et al., 1998; Liu et al., 1998). These factors can bind to the DRE/CRT regulatory element CCGAC (Baker et al., 1994). This element is mostly found in the promoter region of *COR* genes (Medina et al., 2011). There is high similarity (>85%) among the three *CBF1-CBF3* genes, suggesting that they may have originated from the same gene (Gilmour et al., 1998; Medina et al., 1999). Overexpression of *CBF1*, *CBF2*, and *CBF3* substantially increased the frost resistance of plants and significantly induced the expression of *COR* genes in plants (Liu et al., 1998). In this study, we identified only *CBF3* (*DREB1A*) but did not detect expression of *CBF1* (*DREB1B*) and *CBF2* (*DREB1C*). Researchers have found similar results in tomato. Only *CBF1* expression was found to be induced by cold stress, and only three *CORs* were involved downstream, much fewer than the 30 found in *A. thaliana*. The findings also suggested that the cold stress response pattern of tropical plants may be different from that of conventional plants (Zhang et al., 2004). In *P. auriculata*, also a standard tropical plant, only *CBF3* and two *CORs* (*ERD4, ERD7*) were detected at low temperatures, which may contribute to the fact that tropical plants are much less tolerant to low temperatures than conventional plants. Indeed, although CBF, now known to be the most important transcription factor involved in the response to cold stress, has also been demonstrated to be present in a wide range of plants, its expression varies considerably among different species, especially those from the tropics (Carvallo et al., 2011). This unique phenomenon in tropical plants may represent a breakthrough in breeding for improved cold tolerance.

Overexpression of *PaDREB1A* in *A. thaliana* not only significantly enhanced freezing tolerance but also protected the photosynthetic system and enhanced the ROS scavenging mechanism in the plants at low temperatures. In addition, *CORs* of *A. thaliana* could also be upregulated by PaDREB1A. These experimental results were consistent with expectations and similar to those of other studies (Cominelli et al., 2005; Nakashima et al., 2009). In addition, *CAT1* also appeared to be upregulated, which could be an important reason for the increased activity of the protective enzyme *CAT*. However, in both transgenic strains, for *COR15A*, only DRE-13 showed upregulated expression, while DRE-11 showed almost the same expression as the WT plants. Moreover, the cold tolerance of DRE-13 also seemed to be higher than that of DRE-11, with differences appearing among the different transgenic strains. We speculate that this difference may have been caused by the difference in the gene insertion location, where in some strains, the insertion site caused partial disruption of the original gene function. Notably, overexpression of *PaDREB1A* in *A. thaliana* resulted in higher cold tolerance. However, we also found slow growth and smaller leaves in most transgenic lines compared to WT plants (Figure 5). This trait does not seem to be correlated with the expression level of *PaDREB1A* if the transgenic strains are likely to show this trait. Several studies have shown that overexpression of *CBF* leads to plant dwarfing and delayed flowering (Gilmour et al., 2004; Park et al., 2015), but in this study, only slow growth was observed, and flowering time was not affected. It has been shown that overexpression of *CBF1* can activate a decrease in gibberellic acid (GA) content in plants, resulting in a high level of accumulation of the DELLA protein, a negative regulator of the GA signaling pathway, leading to growth inhibition, which can be relieved by exogenous application of GA (Achard et al., 2008). This suggests that this slow growth may be related to the influence of CBFs on hormone metabolism.

In conclusion, we identified *PaDREB1A* as a key transcription factor in *P. auriculata* under cold stress. Overexpression of *PaDREB1A* in *A. thaliana* enhanced freezing resistance, protected the photosynthetic system, and enhanced the ROS scavenging mechanism under cold stress. The enhancement of these cold tolerance-related indicators may be achieved by PaDREB1A through the activation of downstream *CORs* and other related genes.

## Data Availability Statement

The datasets presented in this study can be found in online repositories. The names of the repository/repositories and accession number(s) can be found at: PRJNA797114.

## Author Contributions

ZZ: data curation. WL: formal analysis and writing—original draft. SG: methodology. TL: project administration. YD: validation. LJ and SG: writing—review and editing. All authors contributed to the article and approved the submitted version.

## Funding

This work was supported by the Sichuan Science and Technology Program (2021YFYZ0006).

## Conflict of Interest

The authors declare that this research was conducted in the absence of any commercial or financial relationships that could be construed as a potential conflict of interest.

## Publisher’s Note

All claims expressed in this article are solely those of the authors and do not necessarily represent those of their affiliated organizations, or those of the publisher, the editors and the reviewers. Any product that may be evaluated in this article, or claim that may be made by its manufacturer, is not guaranteed or endorsed by the publisher.

## Footnotes

^1^ http://smart.embl-heidelberg.de/

^2^ https://blast.ncbi.nlm.nih.gov/Blast.cgi

^3^ https://espript.ibcp.fr/
