# Supplementary material for: Transcriptome Analysis Revealed a Cold Stress-Responsive Transcription Factor, PaDREB1A, in Plumbago auriculata That Can Confer Cold Tolerance in Transgenic Arabidopsis thaliana
Source: Front Plant Sci. 2022 Mar 4;13:760460. doi: 10.3389/fpls.2022.760460 (PMC8931719; doi:10.3389/fpls.2022.760460)
Supplement: Supplementary file 1 [file Data_Sheet_1.docx]

Supplementary Material

# Supplementary Figures and Tables

**Supplementary Table 1.** The primers used in this study

| **NO.** | **Primers** |  |
| --- | --- | --- |
| 1 | c20470.graph_c2-F | TCACACCCACTCCCGCTGTAA |
|  | c20470.graph_c2-R | GTGCTTCTCGTCTGGCTTCTGT |
| 2 | c12606.graph_c0-F | CAACGGCGACGGCATGATCT |
|  | c12606.graph_c0-R | TCGTGGTTCGTGTCCATCTCCT |
| 3 | c16006.graph_c2-F | ACACCAAGCGAATCCACCTCAT |
|  | c16006.graph_c2-R | GCCAGCCTCTTCCTACTCTCAC |
| 4 | c7437.graph_c0-F | GGCGAGAACAGGACGAGATACA |
|  | c7437.graph_c0-R | CGTGGCAGAACCCGTACTCTT |
| 5 | c14344.graph_c0-F | CGCCTCAGTCGGTGATCTACAT |
|  | c14344.graph_c0-R | GCCTCAGCCAGCCTAGCAAT |
| 6 | Derb1A-F1(gene clone) | ATGGACGCCTTCTTATCATACTACC |
|  | Derb1A-R1(gene clone) | TCAAACAGAGTAGCTCCAGAGTGGCAAGT |
| 7 | hyg-F | TGTAGTGTATTGACCGATTCCTTGC |
|  | hyg-R | GTTCGACAGTGTCTCCGACCTGAT |
| 8 | PaDreb 1a-F | CAATCACAACAACCCTAC |
|  | PaDreb 1a-R | CTCCTTGAACTTCTTCCT |
| 9 | CAT1-F | TATTCTTCGTCCGTGATG |
|  | CAT1-R | TCCTGAATGTGAGACTTG |
| 10 | ERD4-F | ATGCTCATCCTCACAATC |
|  | ERD4-R | CCAGCCTAAACCAAAGTA |
| 11 | ERD7-F | CGCTGTTCTCGCTAGTGTCTCC |
|  | ERD7-R | ACCTGCTTCCTTCGCTTCCTCT |
| 12 | COR15a-F | AAGAGGCATTAGCAGATG |
|  | COR15a-R | TCCTTTCTCCTCCACATA |
| 13 | 18s-F | CAACCATAAACGATGCCGA |
|  | 18s-R | AGCCTTGCGACCATACTCC |

**
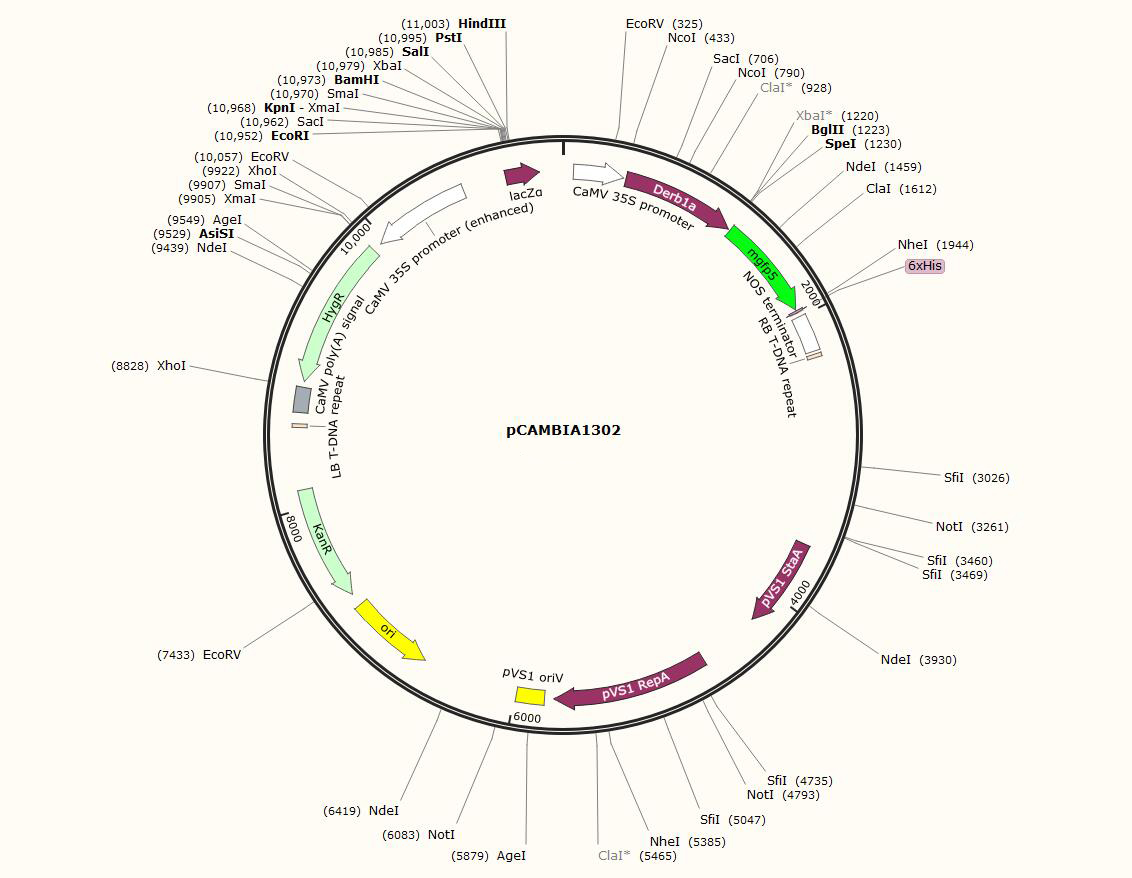
**

**Supplementary Figure 1.** The *pCAMBIA1302-PaDREB1A-EGFP* overexpression vector


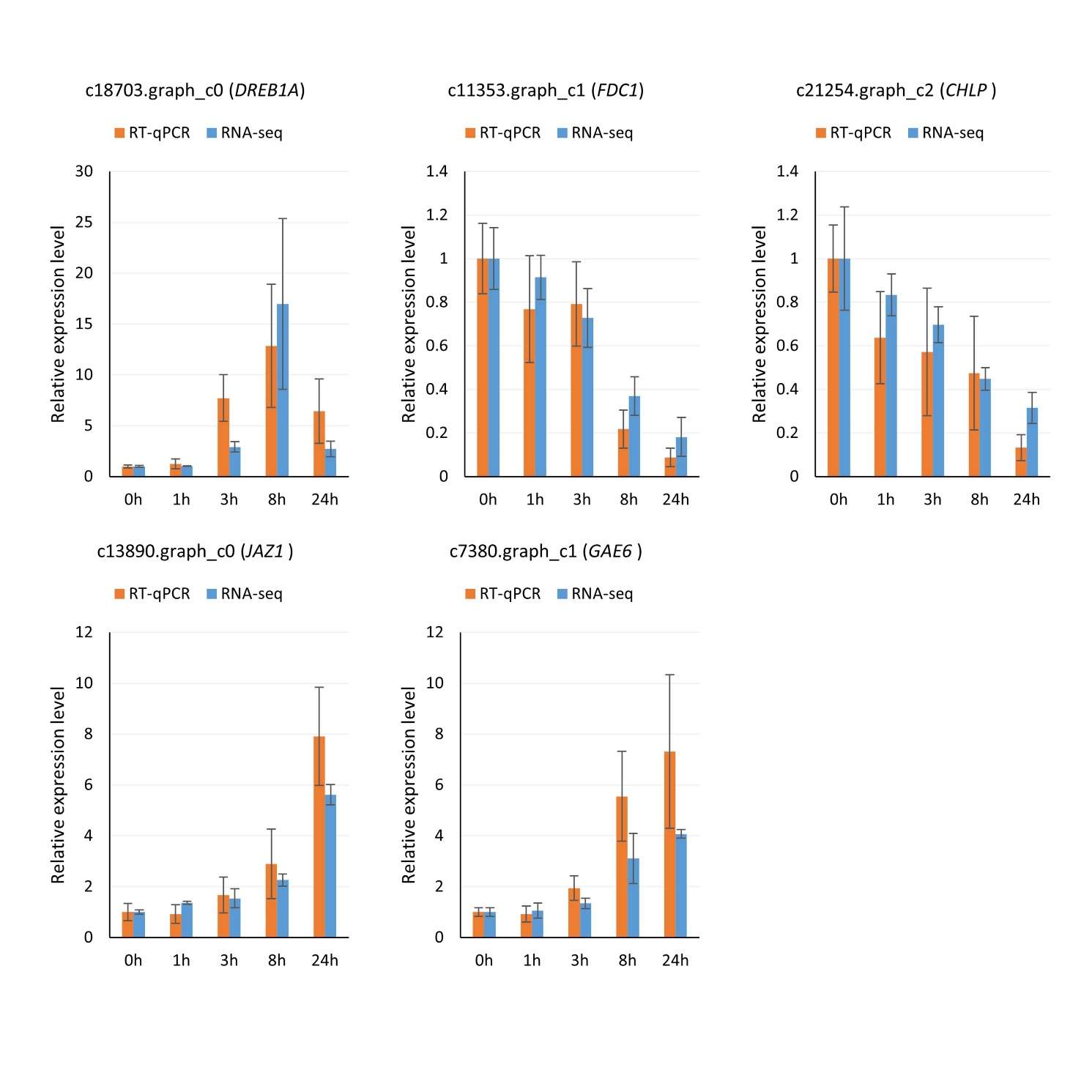


**Supplementary Figure 2.** The relationship between RT-qPCR and RNAseq fold change results


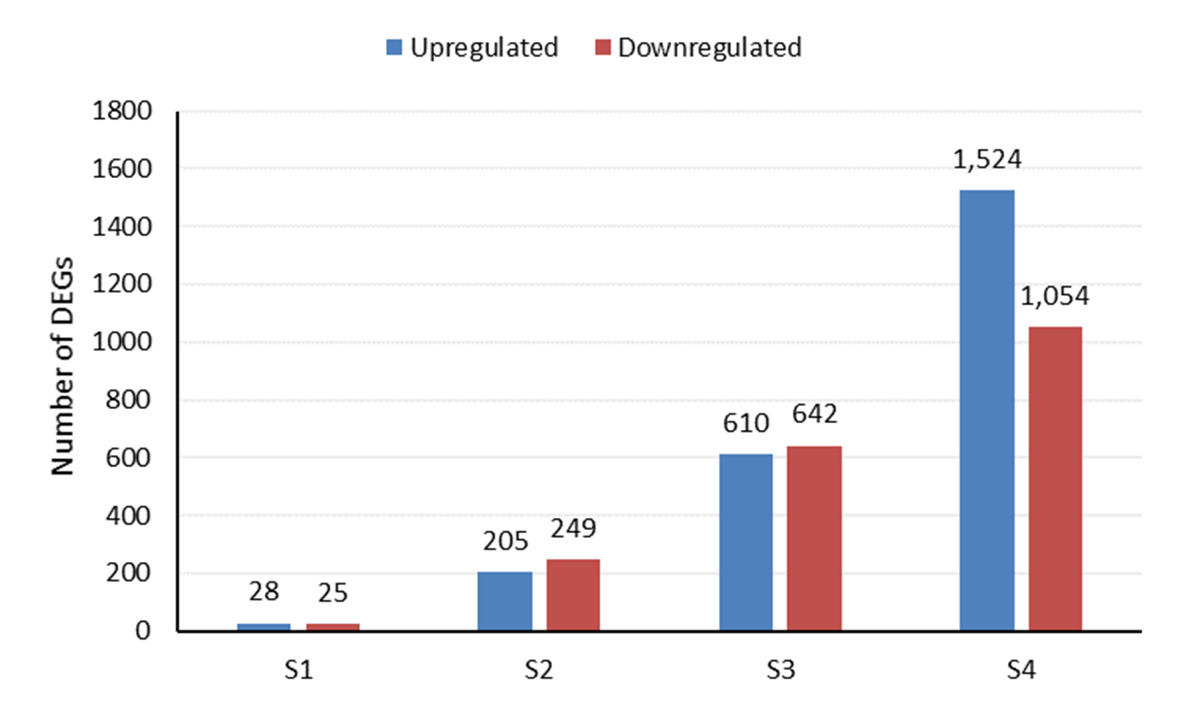


**Supplementary Figure 3.** Differential expression analysis after different cold stress treatment times of *P. auriculata.*


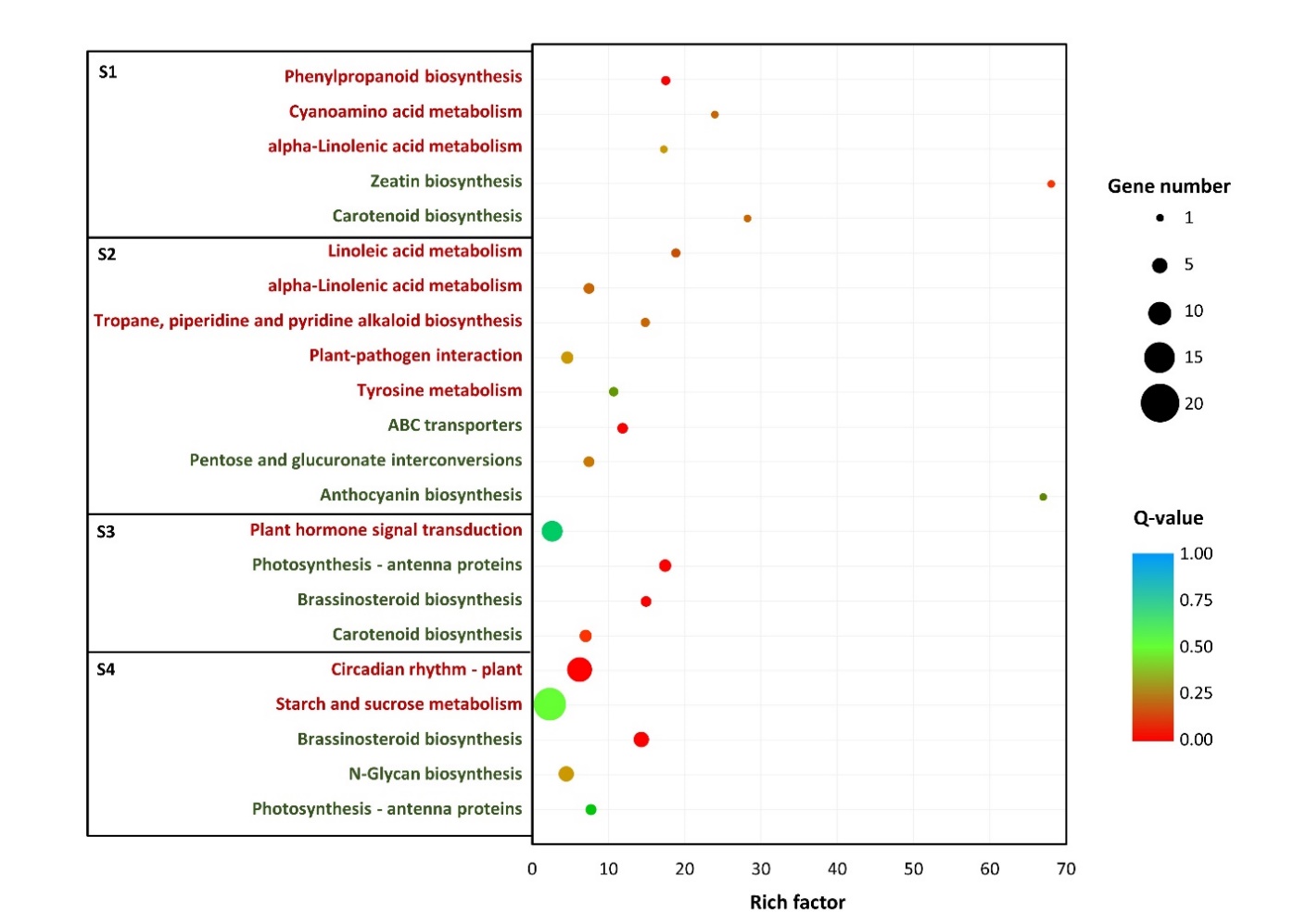


**Supplementary Figure 4.** Scatter plot of enrichment factors in the KEGG pathways of differentially expressed genes after different cold stress treatment times of *P. auriculata*. Red text represents upregulated pathways and green text represents downregulated pathways


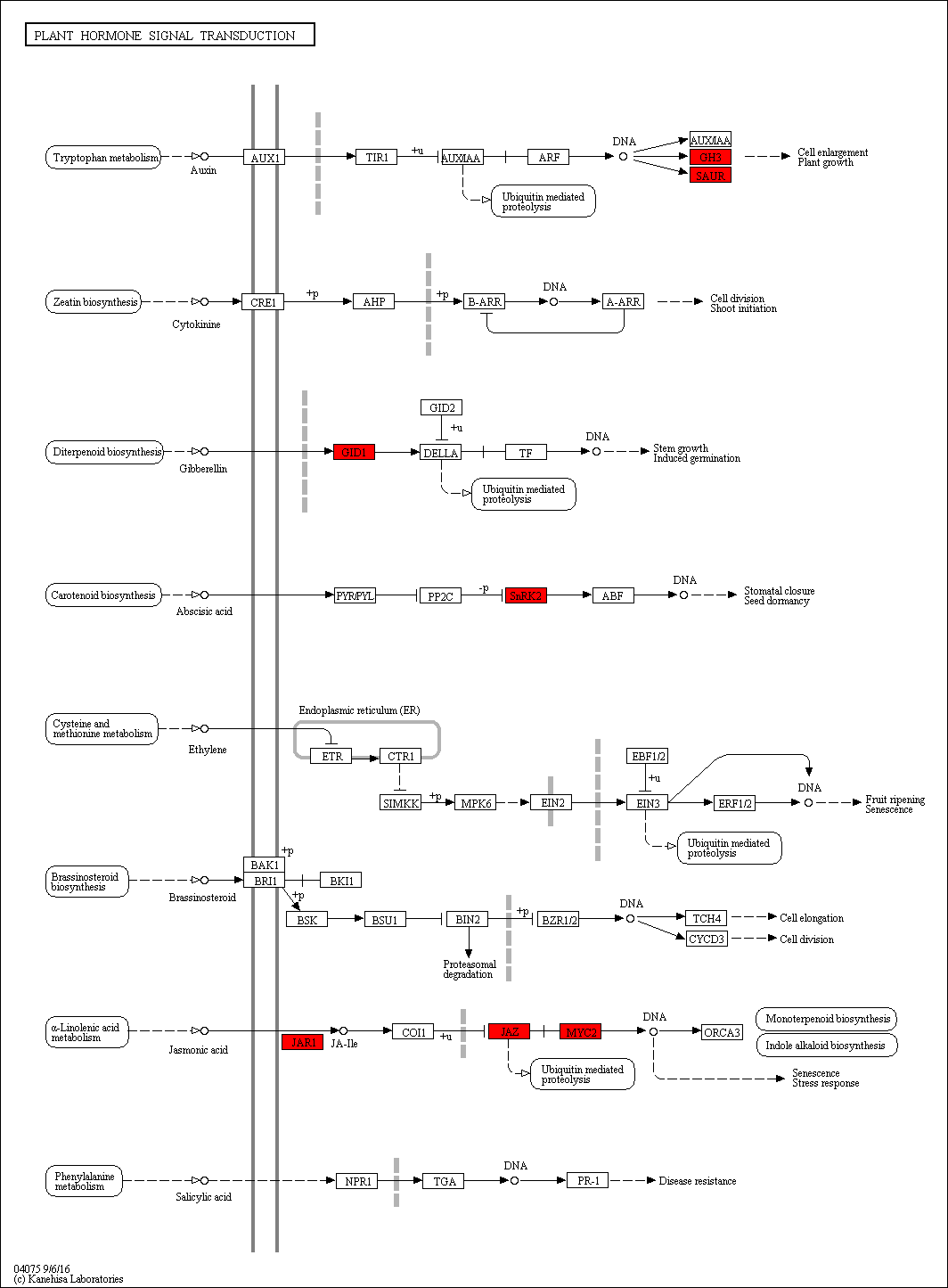


**Supplementary Figure 5.** Annotation of plant hormone signal transduction pathway (ko04075)


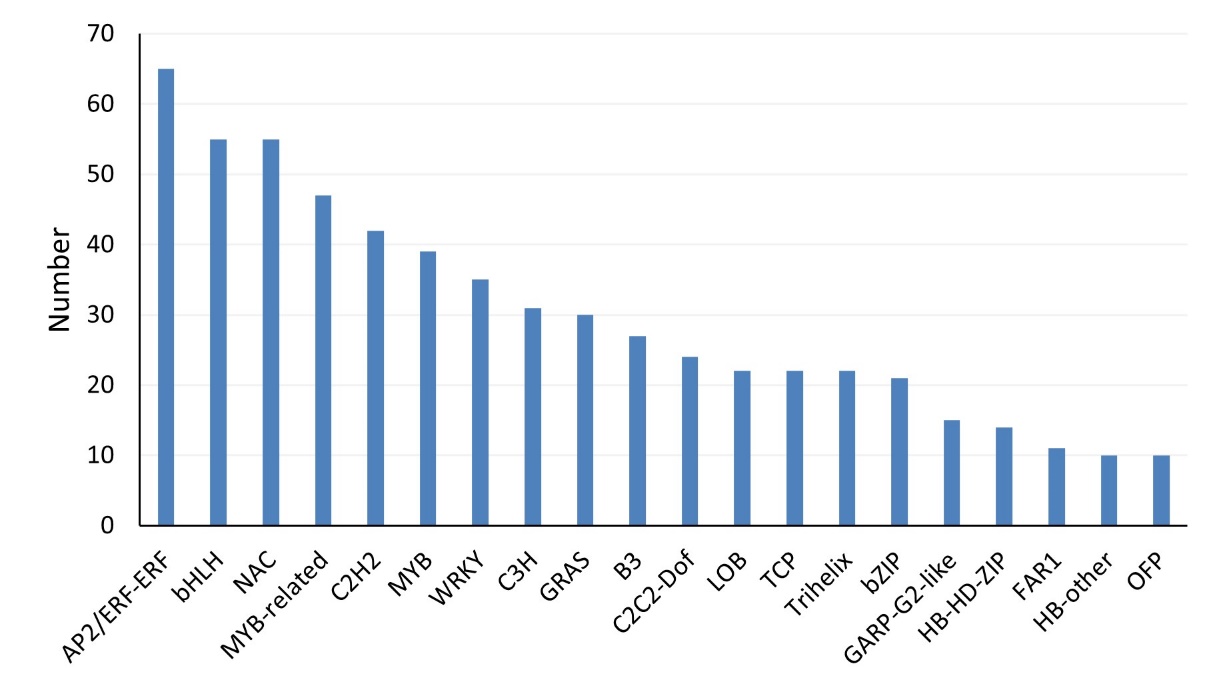


**Supplementary Figure 6.** Transcription factor family prediction after cold stress treatment of *P. auriculata*.


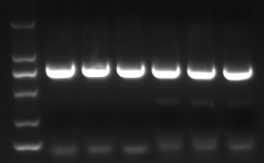


**Supplementary Figure 7.** PCR amplification results of *PaDREB1A* gene


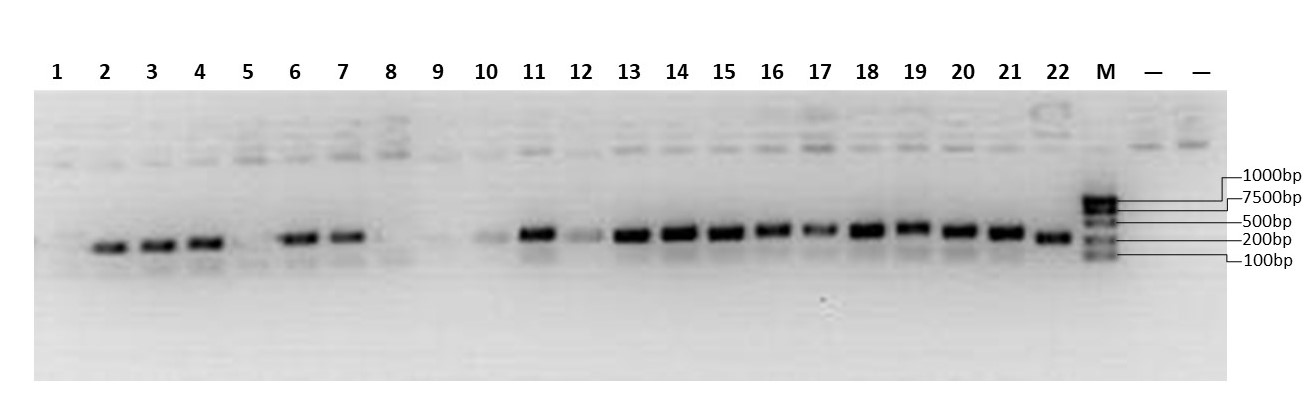


(A)


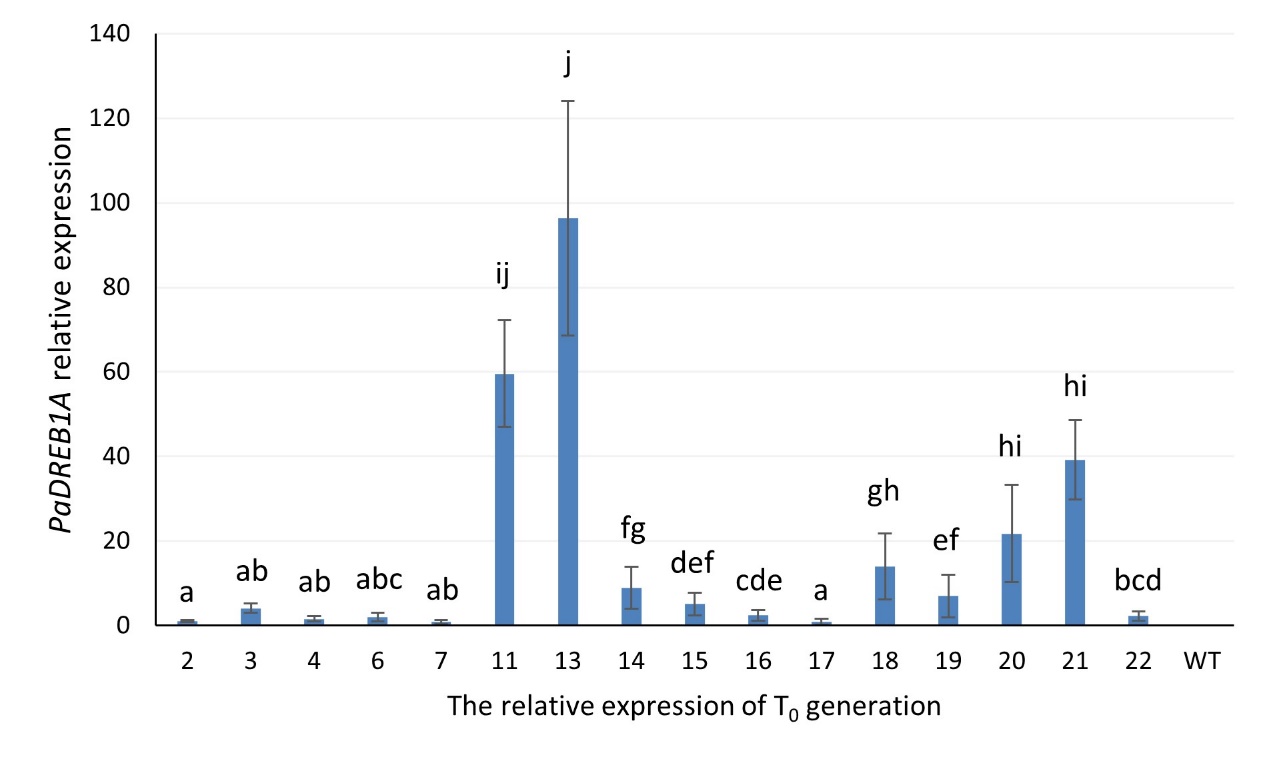


(B)

**Supplementary Figure 8.** Identification of *PaDREB1A-*overexpressing plants of the T0 generation. (A) Electrophoresis results of PCR in T0 transgenic plants (the PCR product of the target gene was approximately 380 bp). (B) Relative expression level of *PaDREB1A* in T0 transgenic plants. Means with the same letters are nonsignificantly different by different transgenic lines.


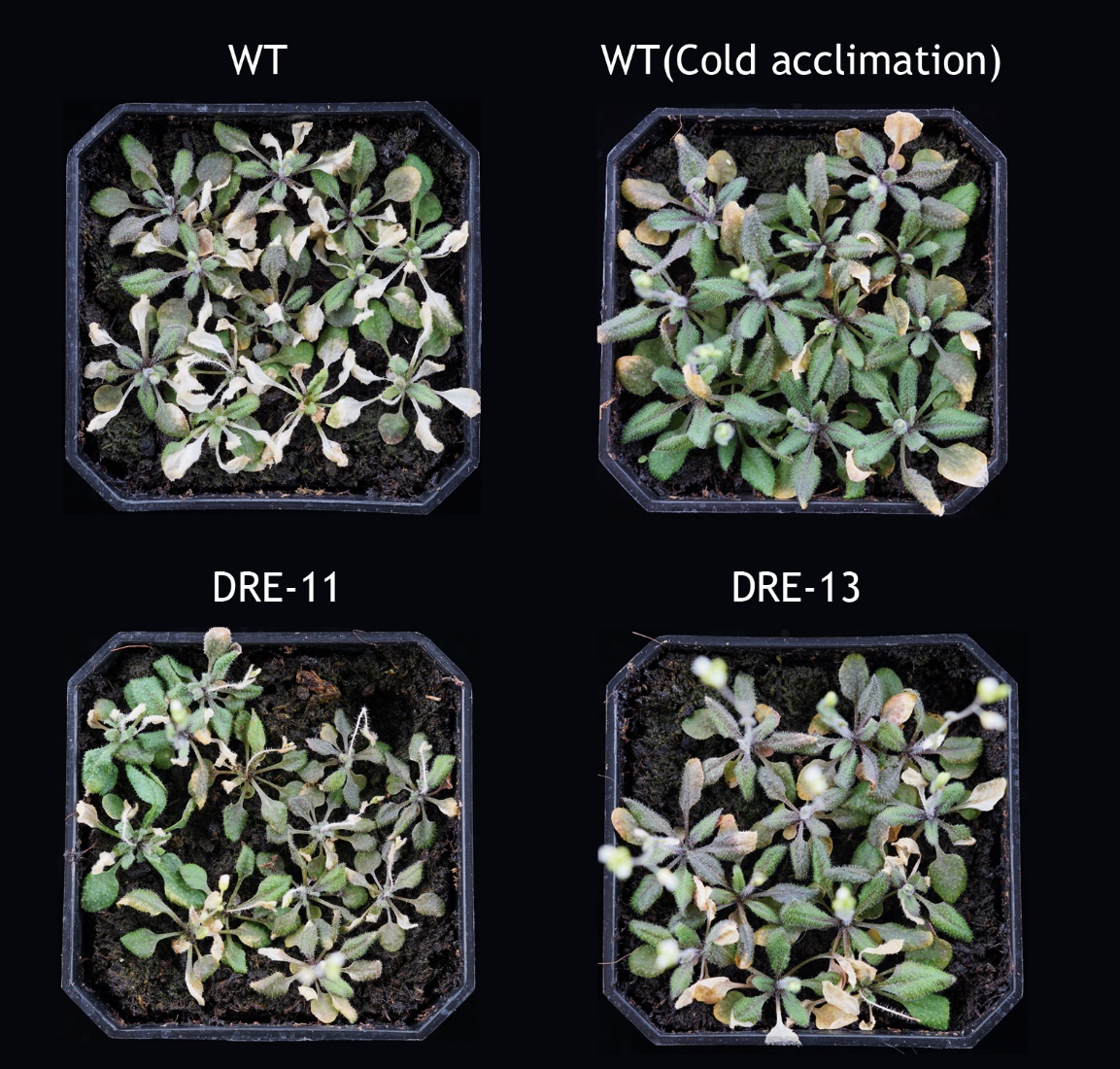


**Supplementary Figure 9.** Cold stress analysis of *PaDREB1A* transgenic lines and WT plants after 72 hours of recovery


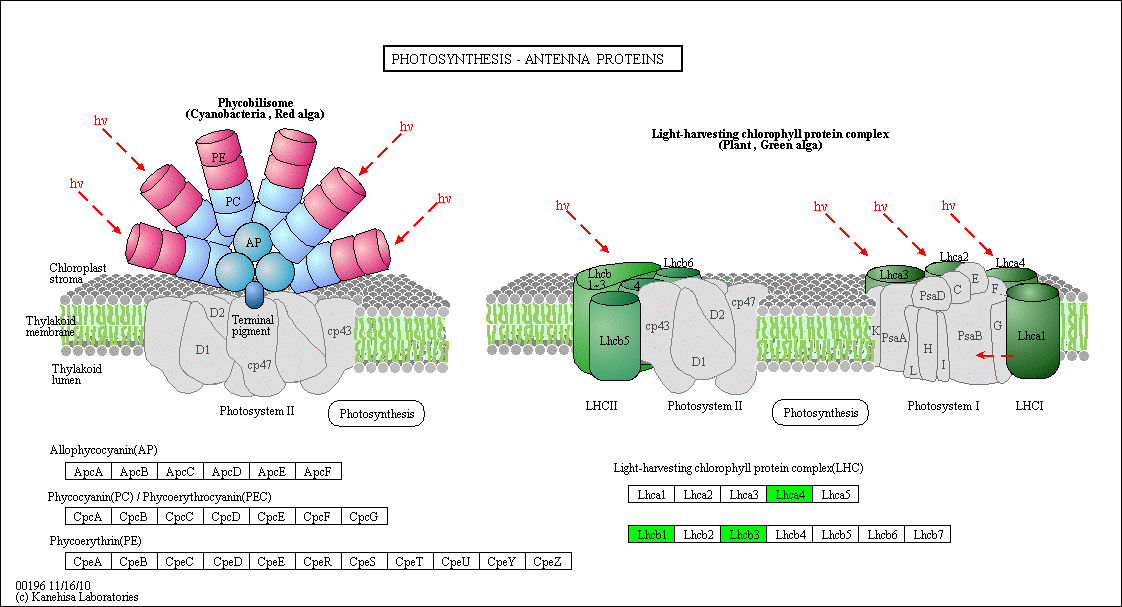


**Supplementary Figure 10.** Annotation of photosynthesis-antenna proteins pathway (ko00196)
